# 19144 Effect of Mesalamine on Metabolic Syndrome risk factors in Ulcerative Colitis Patients: A Retrospective study

**DOI:** 10.1017/cts.2021.752

**Published:** 2021-03-30

**Authors:** Eliseo Castillo, Graziella Rangel Paniz, Fray Arroyo-Mercado, Christina L. Ling, Harry Snow, Eunice Choi

**Affiliations:** University of New Mexico Health Sciences

## Abstract

ABSTRACT IMPACT: Currently, there are no medications to treat metabolic syndrome and our research sheds light on a potential therapeutic that could prove beneficial for this disease that affects one-third of the US population. OBJECTIVES/GOALS: Our goal was to determine the role of the GI tract in MetS, specifically how approved GI-directed medications affect metabolic parameters. Thus, we assessed the effects of mesalamine, a common therapeutic utilized to treat mild to moderate UC, on metabolic parameters in comorbid UC and MetS patients METHODS/STUDY POPULATION: This was a retrospective study with data extracted from Cerner’s HealthFacts database across the United States (US). Inclusion criteria included adult patients (≥18 years old) with a diagnosis of UC and at least 3 of the 5 metabolic risk factors which included i) dyslipidemia, ii) low HDL, iii) hyperglycemia, iv) hypertension, and v) increased abdominal obesity as determined by elevated BMI. A total of 6197 patients across the US between the years of 2007 and 2017 were included. We pulled patients who had a mesalamine prescription within +/- 7 days of an encounter in which they were diagnosed with UC (index date) and the closest values to 3 and 12 months after the index date. Mean age for patients was 53.8 ±19.9, with predominance of female sex (52.9%) and white race (78.0%). RESULTS/ANTICIPATED RESULTS: There was an observed reduction in BMI, fasting glucose, and increase in HDL levels post start of mesalamine treatment along with a decrease in inflammatory markers (ESR and CRP) (p<0.001). DISCUSSION/SIGNIFICANCE OF FINDINGS: The GI tract contributes to numerous disorders associated with metabolic dysfunction. Our retrospective analysis revealed mesalamine treatment in comorbid UC and MetS patients improved metabolic parameters, providing evidence that targeting the GI tract in these individuals potentially improves dysregulated metabolic processes.

